# Permeability-driven selection in a semi-empirical protocell model: the roots of prebiotic *systems* evolution

**DOI:** 10.1038/s41598-017-02799-6

**Published:** 2017-06-09

**Authors:** Gabriel Piedrafita, Pierre-Alain Monnard, Fabio Mavelli, Kepa Ruiz-Mirazo

**Affiliations:** 1 Wellcome Trust Sanger Institute, Hinxton, Cambridge UK; 2 Department of Biochemistry and Cambridge Systems Biology Centre, University of Cambridge, Hinxton, UK; 3 Institute for Physics, Chemistry and Pharmacy, University of Southern, Denmark, Denmark; 4 Department of Chemistry, University of, Bari, Italy; 5 Biofisika Institute (CSIC, UPV-EHU), 48940 Leioa, Bizkaia, Spain; 6 Department of Logic and Philosophy of Science, University of the Basque Country, Donostia-San Sebastian, Spain

## Abstract

The origin-of-life problem has been traditionally conceived as the chemical challenge to find the type of molecule and free-solution reaction dynamics that could have started Darwinian evolution. Different autocatalytic and ‘self-replicative’ molecular species have been extensively investigated, together with plausible synthetic pathways that might have led, abiotically, to such a minimalist scenario. However, in addition to molecular kinetics or molecular evolutionary dynamics, other physical and chemical constraints (like compartmentalization, differential diffusion, selective transport, osmotic forces, energetic couplings) could have been crucial for the cohesion, functional integration, and intrinsic stability/robustness of intermediate systems between chemistry and biology. These less acknowledged mechanisms of interaction and molecular control might have made the initial pathways to prebiotic systems evolution more intricate, but were surely essential for sustaining far-from-equilibrium chemical dynamics, given their functional relevance in all modern cells. Here we explore a protocellular scenario in which some of those additional constraints/mechanisms are addressed, demonstrating their ‘system-level’ implications. In particular, an experimental study on the permeability of prebiotic vesicle membranes composed of binary lipid mixtures allows us to construct a semi-empirical model where protocells are able to reproduce and undergo an evolutionary process based on their coupling with an internal chemistry that supports lipid synthesis.

## Introduction

The origin-of-life field is primed to become a *systems* domain^1^. Most prebiotic chemistries explored to date have been limited to follow the widely accepted strategy of determining which biomolecules came first (cf. proteins *versus* DNA/RNA), the evaluation criteria relying on the abiotic plausibility and the catalytic or template activity displayed by one or another type of molecule^2, 3^. However, this approach misses the crucial point that life, in all its manifestations, comprises a broad *diversity* of molecules in interaction. Recent advances in organic abiotic synthesis^4^ bolster the possibility that relatively high levels of biomolecular diversity were present on the Earth from very early stages (even at a stage where only the first *precursors* of current biopolymers were present), suggesting new scenarios for the stepwise acquisition of more complex (biological) traits^5^. This alternative view implies a shift of focus from *molecular* to *systems* level phenomena in chemistry^6^: from investigating the structural, dynamic or evolutionary properties of a population of molecules of a certain kind, to enquiring about how molecules of different kinds may interact and get functionally coupled, becoming part of more intricate networks and supramolecular assemblies–with correspondingly more intricate structural, dynamic and evolutionary properties. Thus, beyond the mechanisms and kinetics of the reactions involved, one needs to take also into account material constraints related to the *spatial organization* of those reactive processes, like the presence of dynamic interfaces, diffusion barriers, semi-permeable compartments, gradients or osmotic forces.

Research on protocellular systems, incremental in the last decades^7, 8^, is the most promising avenue to tackle this wider range of aspects, providing a natural link with the simplest biological organisms–prokaryotic *cells*. In this context, a series of growth mechanisms and competition dynamics have already been described with simple lipid compartments as key players^9–14^, demonstrating that the problem of prebiotic evolution is not restricted to the emergence of molecular heredity mechanisms and kinetic control over chemical reactions. A physical mechanism as simple as osmotic pressure can favour the growth of those prebiotic vesicles encapsulating more active metabolisms^11^. Competition among vesicles with membranes of mixed composition, driven by different lipid adsorption/desorption rates, has also been reported^13^, where moderate amounts of phospholipid in the bilayer enhanced fatty-acid membrane growth. Unlike osmotic swelling, this latter pathway does not hinder the protocell division step, although it would require an underlying chemistry supporting the synthesis of the complex phospholipid and might involve some dilution of the internal contents. Osmotic differences between the inner and outer aqueous environment of a vesicle will in any case be critical for its steady growth and eventual reproduction^15^. Given these considerations, how can a certain evolutionary capacity be achieved, while preserving a robust and harmonious protocell development?

In this paper we argue for the importance of considering chemical and osmotic effects together, through an adequate coupling of membrane and proto-metabolic dynamics. More precisely, we show how the presence of an internal chemistry that produces lipid components spontaneously taken up by the membrane (mimicking endogenous lipid synthesis, a fundamental biological feature under investigation in diverse labs)^16, 17^ can simultaneously (i) enhance metabolic activity and (ii) lead to faster reproduction cycles of the protocells, thereby increasing their evolutionary potential. The key factor driving the process is the higher permeability that membranes with heterogeneous lipid compositions tend to display (as compared to pure ones). We first report a series of experimental results (leakage assays) making use of vesicles with a mixed-lipid membrane, which complement previously published data on similar heterogeneous compartments^12, 18^. Then, we explore the often neglected, functional and evolutionary implications of membrane permeability and its composition-induced variability: our empirical data were transformed into a permeability curve, used to carry out computer simulations of the complex dynamic behaviour that would emerge when an endogenous chemistry changed the membrane composition of early protocells. Two hypothetical proto-metabolic reaction networks were modelled (a ‘heterotrophic’ version and an ‘autotrophic’ one) and the results compared, highlighting the advantages and disadvantages that the encapsulation would bring about in each case.

## Results

### Non-monotonical variation of membrane permeability as a function of composition

The problem of accessibility of nutrients and release of waste products (fundamental in any study of compartmentalized, non-equilibrium chemistry) becomes especially relevant when dealing with protocells, which were likely formed by heterogeneous mixtures of self-assembling and reacting molecular species. In this context, our first motivation was to assess how the physical properties of model membranes of diverse composition could change during the initial stages of their development (i.e. as prebiotically available lipids were progressively substituted by more elaborate, *de novo* synthesized ones) because those trends would likely determine the dynamic robustness of the incipient protocellular systems, as well as their evolutionary potential^13^. To this aim, *in vitro* experiments were conducted using several mixed-composition lipid vesicles, representing different transition points along the hypothetical timeline of ancestral evolutionary changes leading to biomembranes^19, 20^. Taking dodecanoic (lauric) acid (LA) as a representative of plausible prebiotic amphiphiles, vesicles were prepared from binary combinations of this fatty acid and a bulkier derivative, either glycerol monolaurate (LA/GML vesicles) or the phospholipid species dilauroylphosphatidylcholine (LA/DLPC vesicles), at various molar ratios. There are various ways in which prebiotic amphiphiles may develop into more complex lipids (e.g., through hydrophobic chain elongation), inducing changes in the properties of the corresponding membranes, but here we decided to focus on polar head conversion (see Discussion).

**Figure 1:**
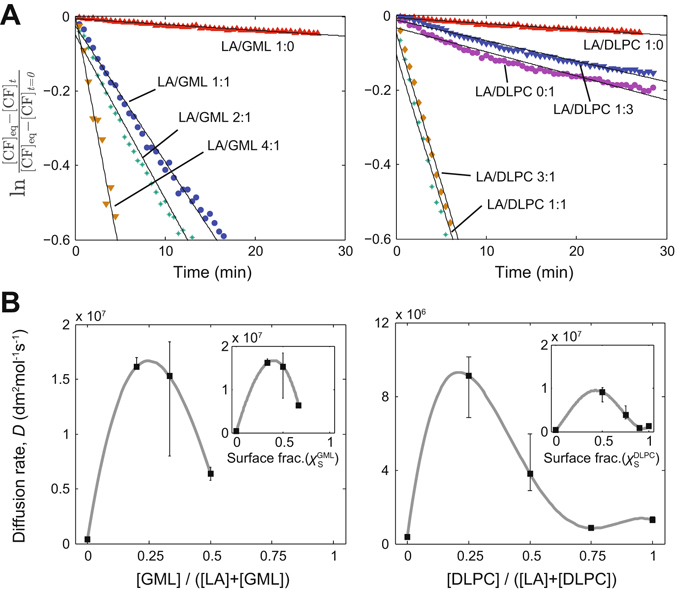
Solute permeability through model protocell membranes as a function of the relative abundance of fatty acid derivatives. (**A**) Release of encapsulated carboxyfluoresceine (CF) at 45 °C from diverse mixed-composition membrane vesicles made of binary mixtures of LA/GML (left panel) or LA/DLPC (right panel) at different lipid molar ratios. Linear regression fits with R^2^ ≥ 0.96. (**B**) Diffusion rates show a non-linear dependency with the lipid molar fractions, with the highest permeability values obtained for heterogeneous membranes. Similar vesicular radii *r* of ca. 200 nm (consistent with the monodisperse extruded population) and membrane thickness values of λ = 3 nm were assumed for the calculations. Inserts: Diffusion rates as a function of the relative membrane surface area covered by the fatty acid derivative species (inferred from the approximate amphiphile molecular head areas: 0.2 nm^2^ for LA; 0.4 nm^2^ for GML; 0.6 nm^2^ for DLPC). Grey curves correspond with exact-matching polynomial fits. Data shown as mean ± SD (n ≥ 3).

Vesicle stability was analyzed both in terms of the critical amphiphile concentration for vesicle formation (*cvc*) and pH sensitivity, confirming that it is highly dependent on the lipid composition: vesicle robustness increased with the content in one or the other fatty-acid derivative (SI Text, Table S1 and Figs S3, S4), in clear agreement with the stabilization effects reported for similar lipid derivatives^18^. In turn, permeability to small molecules was quantified through solute release assays upon encapsulation of a standard fluorophore (carboxyfluoresceine, CF) (Fig. 1A and Fig. S1). Interestingly, unlike stability trends, permeability values did not follow a monotonous trend with the lipid molar ratios, but showed a markedly non-linear dependence (Fig. 1B), reaching a maximum for intermediate lipid compositions (molar ratios ~ ¼ - ½) (SI Text, Table S2 and Fig. S2). This applied both to LA/GML and LA/DLPC systems, where the most permeable conditions were indeed the heterogeneous ones (Fig. 1B, inserts), in line with previous work on vesicles of prebiotic-lipid mixtures^12^ (see Discussion). Altogether, these empirical results allowed us to make a quantitative estimate about how some of the key physical properties of these membranes would vary as their composition changed over time, in a manner tentatively corresponding to various stages of ancestral lipid evolution.

### Dynamical constraints from the early interplay between metabolisms and compartments

**Figure 2:**
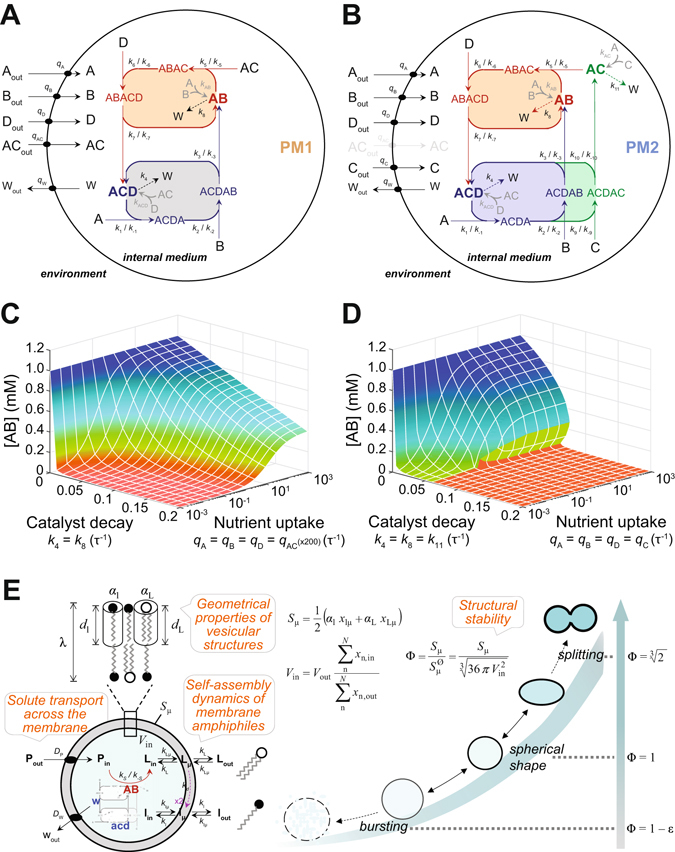
Coupling between primitive compartments and metabolisms: protocell modeling assumptions and physical-chemical constraints. (**A**,**B**) Two minimal models of protometabolism are considered: PM1 (**A**), which is formed by two mutually-promoting catalytic cycles driven by the AB and ACD species; and PM2 (**B**), containing one extra reinforcing cycle leading to the AC species. Both of them rely on the uptake of the corresponding energy-rich precursors from the environment and on rudimentary catalysts of limited lifespan (decay reactions shown as dashed arrows). (**C**,**D**) The build-up of metabolites within a membrane compartment, represented by the steady-state concentration of the species AB, is sensitive to both the decay rates of catalysts and the accessibility to precursors (i.e. nutrient permeability through the membrane). These factors influenced PM1 (**C**) and PM2 (**D**) differently, depending on the needs and costs of each metabolic network architecture. (**E**) The L lipid species was assumed to be synthesized internally from a given diffusible precursor P, in a reaction catalyzed by AB. L contributed to increase the overall protocell surface area S_μ_ and displaced (more or less efficiently, depending on *k*_*d*_) the naturally-occurring l lipid molecules within the membrane bilayer. In addition, some end-products of the protometabolic activity (the permeable *w* species and the totally impermeable *acd* species) tend to accumulate within the protocell, inducing volume growth (through a simulated water inflow–to keep the isotonic condition). The actual balance between vesicular surface area and internal volume *V*_in_ determined the protocell stability and, ultimately, its propensity to undergo splitting or bursting (see main Text and SI).

Our next objective was determining the functional and evolutionary effects that the progressive association of primitive compartments with an underlying chemical network could have caused in a prebiotic context. In a first approximation to the problem, we focused on the process of *encapsulation* itself, as a potential source of direct interactions between early membranes and metabolism dynamics. Nutrients or precursors were distinguished from other metabolites because the former were the only ones present in the environment, and the chemistry was assumed to take place just within the encapsulated reaction volumes. Two different hypothetical models of proto-metabolism were considered: (i) a simple autocatalytic system based on two cross-feeding reaction cycles (designated PM1; Fig. 2A), inspired by Kauffman’s theoretical work^21^; (ii) a slightly more intricate self-producing network consisting of three intertwined catalytic cycles, inspired in Rosen’s^22^ ‘M-R systems’, already studied in the past^23, 24^ (PM2; Fig. 2B). Despite their structural similarities (both reaction schemes rely on the acquisition of external energy-rich precursors across the compartment boundaries and involve cross-catalytic cycles of condensation to achieve *self*-production), PM1 was chosen as a simplified ‘heterotrophic’ model of metabolism, while PM2 was conceived as an alternative (slightly more complex) ‘autotrophic’ version (for further details see SI). Protometabolic architectures based on direct autocatalytic cycles (like the ones present in Ganti’s chemoton model^25^ – see also ref. 26) were discarded in favour of reaction networks that could reach non-trivial stationary states in the absence of compartments.

The two proto-metabolic models were parameterized and simulated under equivalent conditions (see SI) to facilitate direct comparison. The dynamical constraints for their development in compartmentalized conditions were initially explored by steady-state analysis (Fig. 2C,D and SI). The concentrations of the internal metabolites (as well as the metabolic fluxes) proved to be sensitive both to the rates of nutrient uptake and internal catalyst decay. That is, functional proto-metabolisms were found to be only compatible with conditions in which catalysts were moderately stable and the accessibility of precursors was not excessively hindered by the compartment boundary. As pointed out elsewhere^24^ the severity of these restrictions is always relative to the general timescale of the internal reaction network. Up to this level of analysis, time could be kept in arbitrary units *τ*; however, once the experimental range of membrane permeability values (Fig. 1) was introduced, we were forced to consider timescales not smaller than hours (for chemistries that could in practice be developed within this type of primitive compartments, avoiding ‘self-suffocation’–see SI). Although both models showed similar global dynamic trends, interestingly PM2 behaved as a bistable system (Fig. 2D, differently colored surfaces) in contrast with PM1’s monostability (Fig. 2C). This suggests that a relatively minor increase in the complexity of a metabolic network could nonetheless confer it higher plasticity under different circumstances. Such observation could provide a possible explanation for the stepwise development of increasingly complex reaction architectures.

In a second phase, we explored the possibility of a mutual *coupling* between metabolism and membrane, and how this complementary relationship (so prevalent in contemporary cells) could have begun during early origin-of-life stages, by means of an endogenous synthesis of membrane components. We considered the scenario where a novel lipid species L, different from the naturally occurring one l, is synthesized by the internal reaction network (Fig. 2E) via a process catalyzed by AB, the most abundant intermediate in both metabolic models (SI Text, Fig. S5). Given the amphiphilic nature of the new components (and their presumably smaller *cvc* value; SI Text, Table S1), it is reasonable to assume that, once produced, the L lipids would be spontaneously inserted into the compartment membrane. This process does not only contribute to the growth dynamics of the compartment, but is also bound to change its physical properties, including its permeability. It follows that the situation involves a whole new set of constraints that feedback on the global protocell dynamics. In order to study realistically the system-level behaviour of such an intricate web of processes, we made use of the computational platform ‘ENVIRONMENT’, designed to simulate the stochastic time behaviour of chemistries that take place within dynamic lipid compartments^27^. In this way, in addition to all the reaction kinetics, important physical properties of the protocell system were also taken into account (Fig. 2E; SI Text, Fig. S6; see also SI Text). In particular: (i) the membrane was modelled as a 3D topologically-closed lipid bilayer, a geometrical structure of precise thickness *λ* and surface area *S*_μ_, determined by the molecular characteristics of its lipid components; (ii) the processes of association and dissociation of the different lipid species to/from the existing membrane were explicitly simulated; (iii) solute diffusion *D*_x_ across the membrane was assumed to be a function of the membrane lipid composition; and (iv) the inner aqueous volume of the compartment (*V*_in_) was considered to respond almost immediately to osmotic pressure differences with the surrounding environment, re-establishing the isotonic condition at each simulation step. Therefore, within this modeling framework^27^, a protocell may adopt different shapes, depending on its actual surface-to-volume relationship. The *reduced surface* (Φ) can be employed as a parameter to define the structural stability of the system (i.e., its viability range), together with the conditions for it to undergo cellular division or osmotic burst (see SI).

**Figure 3:**
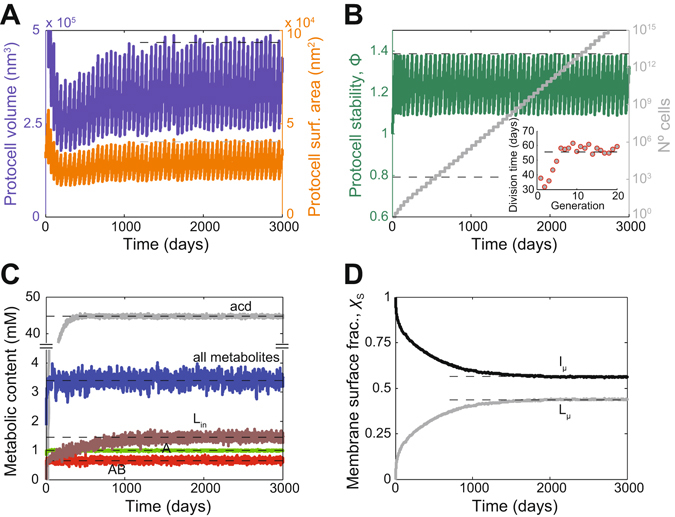
Controlled, stationary reproduction arising from a simple, lipid-synthesizing model protocell. The PM2 chemical reaction scheme is considered, defining lipid synthesis and vesicular properties as given in Fig. 2E. The main dynamical features are displayed. Protocells grow in a harmonious way, with both the internal volume and the surface area increasing at a consistent pace, controlled by the internal metabolism (**A**). A progressively oval-shaped structure evolves, leading to the division condition defined by the stability parameter Φ (**B**). Successive divisions occur from the original mother protocell (jagged pattern), giving as a result an increase of the cell population. Reproduction is achieved with a constant division time (B, insert) and characteristic vesicular sizes (see A), while concentrations of all internal metabolites keep fluctuating around stationary levels (**C**). (**D**) Membrane composition changed as the complex internally-synthesized lipid (L) replaced the original prebiotic one (l). All variables tend to asymptotic values (dashed lines) indicative of the stationary character of the reproduction regime.

The dynamical consequences of an endogenous lipid synthesis were tested separately using PM1 and PM2 as chemical reaction models. Remarkably, irrespective of the underlying metabolic network, the outcome was a stationary reproduction regime for a wide range of parameter values (as stated above: within conditions that keep decay rates of the catalysts and nutrient accessibility at moderate levels) (Fig. 3, SI Text, Fig. S7). Provided that the inner reaction domain suffered a net accumulation of some osmotically-active metabolic end-product (e.g., the *acd* species; Fig. 2E), both the internal protocell volume and the membrane surface area increased, spontaneously conforming to a mutually compatible growth kinetics; hence multiple division cycles from the original mother protocell were obtained (Fig. 3A,B). Contrary to simpler lipid-producing models in which vesicles progressively shrank across successive generations^26^, reproduction proceeded with characteristic, stationary protocell sizes (Fig. 3A, dashed lines) and division times (Fig. 3B, insert), ensuring a sustained increase in the cell population (Fig. 3B, grey line). In addition, despite the continuous, dynamic changes in the shape/size of the compartments, the levels of the internal metabolites remained fluctuating around steady-state values (Fig. 3C), which further supported the robustness of the reproduction regime. The latter result may be caused, at least partially, by the tempered nature of the two internal reaction networks studied in this work (as compared, for instance, with the direct autocatalytic cycles mentioned above^25^), what strongly reduces the risk of osmotic burst. Only when a huge accumulation of metabolic end-products was imposed (setting a permeability constant for the *w* species various orders of magnitude smaller than those for the nutrients), would the protocell collapse (Fig. S7).

### Evolutionary implications of lipid synthesis and membrane permeability changes

In the context of a population of similarly robust, *self*-reproducing protocells, whose dynamic behaviour approaches a stationary division regime, the crucial aspect that will determine the evolutionary outcome at a global scale is the characteristic *division time* displayed by each type of protocell. Under the general assumption that the natural sources of nutrients and of primary amphiphiles are non-limiting, a given type of protocell will spread faster in the population–and eventually outcompete the others–the faster it is able to reproduce. Division time will depend on the system’s growth rate. Although this is a complex trait that requires a balanced metabolic production to suit both protocell surface and volume increase, it is to be affected by the permeability and, ultimately, the composition of the membrane. Thus, we explored the dynamical features emerging from protocells showing diverse degrees of lipid membrane conversion (i.e. diverse l/L ratios for the membrane composition in the stationary state).

**Figure 4:**
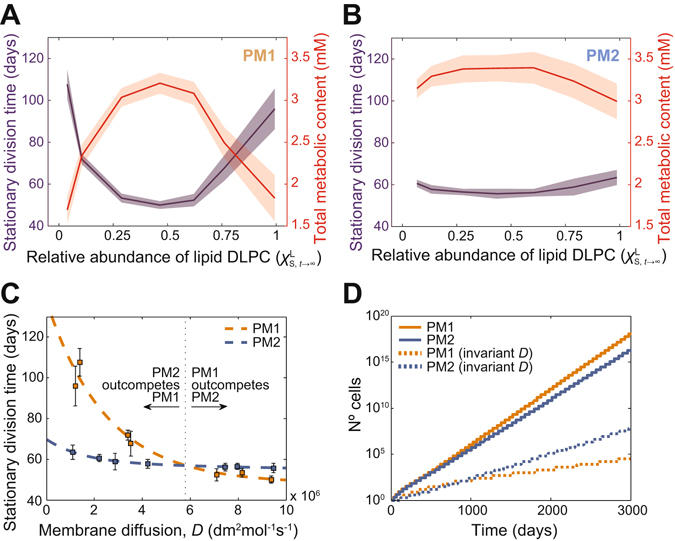
Differential membrane lipid composition triggers selection pressure between model protocells. (**A**,**B**) Lipid-producing protocells attained different division speeds at the stationary reproduction regime depending on their relative capacity to transform the membrane composition and reach heterogeneous lipid mixtures, irrespective of similarly efficient catalytic rates for lipid synthesis (only parameter *k*_*d*_ was varied across simulations). This applied both to PM1-based protocells (**A**) and to PM2-based ones (**B**), the former being more sensitive to changes in their membrane lipid composition. Division times inversely correlate with the stationary concentration levels of the internal metabolites. In both cases, mean values and mean ± SD ranges obtained from 10 consecutive generations at the stationary reproduction regime (t > 2000 days) are shown (solid lines and shaded areas, respectively). (**C**) Differences in membrane diffusion rates are responsible for lipid-composition-driven changes in protocellular division times. Average values and dispersion (error bars) are as in (**A,B**). Dashed lines correspond with exponential fits (R^2^ ≥ 0.96). Details of the metabolic network structure matter in the degree of responsiveness to membrane permeability changes. (**D**) A PM1 type of chemistry may become more advantageous when protocells undergo only partial conversion in membrane lipid composition (χ^L^_S,t→∞_ ≈ 0.5). Time courses related to the population sizes of equivalent protocells (but where no membrane-composition dependent permeability change is allowed) are also included for comparison.

Given the permeability dependence on membrane lipid composition (Fig. 1), protocell division times in the stationary reproduction regime were expected to depend in particular on the ability of protocells to partially transform their membrane composition, hence reaching heterogeneous lipid mixtures. In order to improve our control over the degree of change in the membrane composition, an additional parameter *k*_*d*_ was included in the model (Fig. 2E) helping us tune the final stationary conditions of mixed membrane lipid compositions (Fig. 3D, SI Text, Fig. S8). Otherwise, under most of the investigated conditions, protocells underwent a full conversion of their membrane composition (ending up in the ‘pure L’ configuration), as a result of their internal proto-metabolic activity. It should be remarked that the value of *k*_*d*_ had no influence on the catalytic efficiency of lipid synthesis directly, nor on its net contribution to membrane surface growth (SI). Following the implementation of the composition-dependent permeability (SI Text, Fig. S6), protocells that evolved to have heterogeneous lipid membranes, with higher permeability to nutrients, turned out to divide faster both with PM1 and PM2 (Fig. 4A,B). Indeed, higher permeability values correlated with higher internal metabolic levels and higher yields in surface and volume growth. Consequently, differences in membrane diffusion rates were responsible for lipid-composition-driven changes in protocellular division times (Fig. 4C).

Finally, it is to be noted that the metabolic network structure played an important role in the degree of responsiveness to membrane permeability changes. Despite the similarity of both models, a simpler scheme, PM1, representing the heterotrophic version of proto-metabolism, was more sensitive to permeability changes than PM2, its autotrophic alternative, whose additional and reinforcing reaction loop rendered it less dependent on boundary conditions (Fig. 4C, Fig. S5). The strength of a more elaborate model, such as PM2, is to support higher average metabolite concentrations and faster reproduction kinetics under a wider range of conditions (Fig. 4A,B). But such ability does not *per se* constitute an absolute guarantee for better performance. Once changes in membrane physical properties are taken into account (Fig. 4D), the complementary role of metabolism and membrane in defining protocell evolutionary potential becomes prominent.

## Discussion

Contemporary biomembranes are composed of a large variety of complex lipids, all metabolically synthesized according to the needs of each cell at any given time. Thanks to the presence of other functional ingredients (especially membrane proteins), biological compartments can afford lipids that confer them high stability at the expense of decreasing their overall permeability to hydrophilic solutes. The evolutionary pathway that permitted the emergence of such membranes from their prebiotic precursors is still unknown. But a monotonous transition towards increasingly more impermeable compartments should not be taken for granted, as it has been sometimes done^13^. Protocells probably remained highly heterogeneous in terms of lipid composition from their earliest origins. In fact, there is no solid argument supporting pure or homogeneous prebiotic membranes. Scenarios like the one explored by^28^ seem to be more plausible, as evidenced by the meteorite amphiphile content^29^ or the large spectrum of products in simulated amphiphile syntheses^30^. And it is quite probable that this membrane heterogeneity was later consolidated, because the changes toward modern (biosynthesized) lipids necessarily involved: (i) an increase in the hydrocarbon chain length (one cannot assume abiotic sources of fatty acids longer than 10–12 carbon chains); and (ii) an increase in the size and chemical complexity of the polar head (allowing, among other things, the formation of diacyl structures^20, 31^). Therefore, the degree of heterogeneity in the composition of the membrane itself was possibly not such a significant change, but rather the emergence of an endogenous *control* of that heterogeneity (through the coupling with metabolism).

It is difficult to establish whether (i) preceded (ii), (ii) preceded (i) or both occurred concomitantly. Several studies dealing with early evolutionary changes in membrane lipid composition and derived physical properties have been carried out to date, focusing on diverse liposome compositions^12, 13, 32, 33^, but it is not so easy to disentangle the contributions due to different factors (e.g., changes in lipid polar-head groups *vs* variations in the length of the hydrophobic tails–or in their number) and define a clear transition scheme. Previous reports^18, 34^ already demonstrated that the length of the lipid is a determinant (inverse) factor in the permeability of the corresponding membranes. In this work, we focused on relatively short, fixed-length (C12) membrane lipids, taking lauric acid (LA) as the primary reference, and explored the effects of increasing polar-group complexity (either with molecules of one or two hydrocarbon chains). Both glycerol and glycerophospholipidic derivatives were found to confer greater stability on the LA vesicles, in agreement with other work^18^. However, membrane permeability did not respond monotonically to the abundance of these bulkier amphiphiles, but exhibited maximum values for heterogeneous, intermediate lipid compositions, in line with previous literature reporting increased vesicle permeability associated with curved local membrane deformations^12^. Altogether, this allows us to devise an evolutionary scenario where membranes could have exploited mixtures of amphiphiles of different polar heads *before* acquiring longer lipid components, which would progressively have led to lower solute permeability levels.

The role of solute permeability in early protocell development has itself been subject of numerous speculations^35–38^. This issue could be better addressed, of course, if we had a clearer idea about the repertoire of prebiotically available molecules and the nature and functioning of the first forms of metabolism. Yet, the main objective of this paper was to demonstrate a ‘plausibility window’ rather than a precise account of events; that is, to call attention to an often overlooked line of research. In that sense, the models explored should also be regarded from a general perspective, beyond their specific limitations (on the experimental side, the permeability assays were restricted to one solute, carboxyfluoresceine; at a theoretical level, our treatment was limited to two abstract minimal models of metabolism). They are actually interesting cases from a basic organizational point of view, because they both fulfil the requirements for catalytic self-sustainability^39^, a property which should be shared across different metabolisms^40^. In this context, one should also realize the importance of *integrating* different subsystems into a wider dynamical framework. For instance, when coupled to a dynamic compartment, our proto-metabolic models naturally lead to system growth and reproduction, although they are not, globally speaking, autocatalytic (i.e. each of them would simply ‘maintain itself’ around steady-state values). Similarly, apparently non-functional, chemically inactive end-products (*w* and *acd* species) become key components for linking protometabolic activity to (osmotically-driven) protocell growth. Furthermore, one of the main implications of this work is to establish that relatively small changes in the structure of a metabolic network could have significant phenotypic consequences, if this network is within–or supporting–a wider dynamical process.

In summary, our results confirm the adequacy of a *systems* approach to the question of origins of life, already reflected in the emphasis given to protocellular organization, but more manifest after showing how molecular mixtures add dynamic and structural richness to the basic phenomena. More specifically, lipid variety in the composition of prebiotic membranes could enable high “phenotypic” diversity in primitive cells before the appearance of genetically-encoded transporters. In addition, as described in previous work (both experimental^14^ and theoretical^41^), the non-genetic synthesis of short peptides could have also contributed to functionalize vesicle compartments during the first stages of protocell development. Thus, pre-Darwinian evolutionary processes should constitute the new focus of research in this field. Terms like ‘fitness’ or ‘survival’ ought to be substituted by other features related to the ‘dynamic robustness’ of the supra-molecular assemblies and investigated as the population-level consequences of their various physical-chemical interactions (in an environment with limited resources). Given the complexity of this evolutionary scenario and the great amount of variables that must be taken into account (in particular, to identify ‘system attributes’ with novel selective effects), additional methodologies of analysis have to be developed. These include the different experimental techniques that *systems* chemists are currently implementing for the quantitative analyses of heterogeneous molecular mixtures, like dynamic combinatorial libraries (reviewed in ref. 1), but should also comprise specific tools to study molecular *organizations* with biological relevance. In this context, we consider that the combination of empirical results with theoretical models and simulations–which not only facilitate the interpretation of the former, but also help in the design of new experiments–is fundamental. The semi-empirical methodology we applied in this article to investigate protocell dynamics and evolution is an illustration of this general strategy, bound to provide many other insights in the future.

## Methods

### Materials

All chemicals were obtained from Sigma-Aldrich

### Vesicle preparation

Melted lipids were mixed at the indicated molar proportions in a preheated 100 mM bicine buffer solution at 45 °C (above the melting point of lauric acid (LA) ~ 43 °C), and the pH vesiculation method was followed^18, 42^, so as to facilitate the fatty-acid solubility and maximize the formation of vesicles. A pH = 8.0 was set, close to the p*K*_a_ of LA (Fig. S4). Once formed, all vesicle suspensions were kept at 45 °C throughout the experiments (above the phase-transition temperature *T*_m_ of LA membrane bilayers ~32 °C). In the case of the LA/DLPC mixtures, amphiphiles were first dissolved in a chloroform solution and settled as a thin lipid film upon chloroform evaporation in a desiccator, before resuspension in the bicine buffer.

### Vesicle stability

The *critical vesicle concentration* (*cvc*) was determined following the merocyanine-540 method^18^. The absorbance ratio Abs_570 nm_/Abs_530 nm_ was measured (Lambda 35 spectrophotometer, Perkin Elmer) for serial dilutions of vesicle suspensions at 45 °C, and the total lipid concentration where the inflexion point occurs was selected as the *cvc* value (Fig. S3). Lipid aggregation tests and pH titration were made on lipid dispersions in water (60 mM of total amphiphile concentration): alkaline samples were gradually acidified using aliquots of 0.1 M HCl and turbidity measured as Abs_480 nm_. Vesicle presence was confirmed by epifluorescence microscopy (Eclipse TE2000-S, Nikon) using the lipophilic dye Nile red (e.g. see Fig. S1).

### Permeability measurements

Lipid vesicles were prepared (see above) in a preheated 100 mM bicine buffer solution containing 8 mM carboxyfluoresceine (CF) (total amphiphile concentration in the range 12–22 mM) (SI). Later, vesicles were extruded through polycarbonate filters of 400 nm-pore size, using a small extruder from Avanti Polar Lipids, USA. Non-encapsulated CF was removed by size-exclusion chromatography, on a Bio-Gel A1.5 column (Biorad) pre-equilibrated with 100 mM bicine, pH 8. This equilibration solution contained, in the case of LA or LA/GML vesicles, the corresponding amphiphiles at a concentration close to the *cvc*, to avoid vesicle disruption during the separation. The vesicle-containing fraction was collected and the release of CF monitored over time on a spectrofluorometer (Cary Eclipse, Varian) (Ex: 450 nm/Em: 520 nm) at 45 °C (Fig. S1). The external concentration of CF was determined using a standard calibration curve (Fig. S1) and the permeability calculated from the exponential release rate^24^ (SI).

### Computational analysis

The protometabolic network models were analyzed using MATLAB (SI), and steady-state concentration values obtained by numerical solution of the set of ODEs. Protocell models were developed and their dynamics analyzed stochastically using the platform ENVIRONMENT^27^ (SI). Alternatively, these models were implemented in MATLAB and studied deterministically.

## Electronic supplementary material

SUPPLEMENTARY_INFO_PIEDRAFITA_ET_AL
