# Supplementary material for: Permeability-driven selection in a semi-empirical protocell model: the roots of prebiotic systems evolution
Source: Sci Rep. 2017 Jun 9;7:3141. doi: 10.1038/s41598-017-02799-6 (PMC5466667; doi:10.1038/s41598-017-02799-6)

Supplementary Information

Permeability-driven selection in a semi-empirical protocell model: the roots of prebiotic *systems* evolution.

Gabriel Piedrafita[1,2], Pierre-Alain Monnard[3], Fabio Mavelli[4] and Kepa Ruiz-Mirazo[5,6,*]

[1]Wellcome Trust Sanger Institute, Hinxton, Cambridge CB10 1SA, UK; [2]Department of Biochemistry and Cambridge Systems Biology Centre, University of Cambridge, UK; [3]Institute for Physics, Chemistry and Pharmacy, University of Southern Denmark, DK; [4]Department of Chemistry, University of Bari, Italy; [5]Biofisika Institute (CSIC, UPV-EHU), [6]Department of Logic and Philosophy of Science, University of the Basque Country, Spain.

*kepa.ruiz-mirazo@ehu.eus

Table of Contents

Experimental conditions of the solute release assays S2

Protometabolic modeling S2

Membrane compartment and protocell modeling S5

References S8

Table S1 *CVC values* S10

Table S2 *Permeability coefficients* S11

Fig. S1 *Experimental procedure and solute encapsulation controls* S12

Fig. S2 *Data from encapsulation assays* S13

Fig. S3 *Calculation of the CVC values* S14

Fig. S4 *Vesicle aggregation as a function of pH* S15

Fig. S5 *Metabolite concentration profiles* S16

Fig. S6 *Simulating lipid-composition dependent permeability* S17

Fig. S7 *Robustness of the stationary reproduction regime* S18

Fig. S8 *Time evolution of membrane composition and stationary division times* S19

Experimental conditions of the solute release assays

Different aspects were considered in order to set up the conditions for the solute release measurements. Carboxyfluoresceine (CF) self-quenches at high concentrations (≥ 0.1 mM) and was suitable for the release measurements (Fig. S1). Provided that vesicles were prepared in a relatively concentrated CF solution, the signal from the encapsulated CF remained self-quenched after size-exclusion chromatography, while its growing concentration in the external medium, as a result of the release, could be monitored as a progressive increase in the fluorescence signal over time. However, setting too high an initial concentration of CF could represent a problem to monitor the release if external concentrations reach self-quenching values before equilibration takes place. For that reason, we set an intermediate concentration of [CF] = 8 mM. Considering the vesicle dilution through the chromatography column (≈ 1.25) and assuming as maximum 1% of encapsulation volume, one would estimate a final overall concentration around 0.06 mM, which is still below the self-quenching limit (around 0.1 mM). The fluorescence signal obtained after vesicle disruption with a detergent (Triton X-100 or octyl-*β*-D-glucopyranoside) as well as the standard calibration curve confirmed that equilibration was completed within the non-quenching concentration range.

Lipid concentrations were also chosen to account for the wide range of *CVC* values in the various lipid systems (see Table S1) and ensure that a similar number of vesicles were formed in every sample. We aimed at a 10 mM concentration of vesicle-forming lipid. For instance, in the case of pure-LA preparations, LA was prepared at a total concentration of 22 mM, considering its higher *CVC* value (22 mM lipid yields 10 mM of vesicular lipid, once the dilution factor of the chromatography column is considered, and once the free-monomer concentration is excluded). At the other extreme, LA/DLPC samples were prepared at a total lipid concentration of 12 mM, accounting only for the experimental dilution factor. The chromatographic dilution of lipid vesicles of high *CVC* value becomes problematic, and therefore, in the case of pure-LA vesicles and LA/GML vesicles, the 100 mM bicine buffer used for equilibration and elution was supplemented with the corresponding monomeric amphiphiles at a concentration close to their *CVC*. This protocol was not followed in the case of LA/DLPC vesicles, given their higher stability.

Protometabolic modelling

Rationale of the models

Two main criteria were considered in the conception and choice of the protometabolic cycles: (i) simplicity, balanced with thermodynamic and chemical coherence, and (ii) relevance from a theoretical point of view. The maintenance of both reaction networks depicted in Fig. 2 of the main text, PM1 and PM2, require feeding by external energy-rich precursors (A, B,… species), using these to drive a set of condensation and hydrolysis reactions, and ultimately releasing non-active end-products (w). The irreversibility of this process forces reactions to operate in the direction

established by the energy flow (direction of the arrows in Fig. 2A-B). Interestingly, the molecular composition of all the metabolic species (i.e., the molecular units they are made of) can be explained in terms of the same precursors that feed the system: none of the intermediates is exogenous. This implies that all the given internal compounds could have been originally assembled from a similar chemical environment, following slow and subsidiary, non-catalytic condensation processes (considered in our two models as background reactions; light grey arrows in Fig. 2A-B). In this way, for instance, the AB molecule is a product of the condensation of A and B.

The condensation products AB, ACD (and AC in PM2) are considered as catalytically-active oligomers, and the other intermediaries represent different transient states of their corresponding catalytic cycles (highlighted in different colors in Fig. 2A-B). Remarkably, these cycles are arranged in such a way that the catalysts support each other's formation. A chemical organization like this, where all catalysts can be *self*-produced within the system and need not be externally supplied is commonly referred to as possessing the property of *catalytic closure*[1,2], which has been proposed as a fundamental attribute of life, key in the early development of minimally robust (autonomous) prebiotic systems[3].

The type of organizational closure just mentioned should not be confused with thermodynamic closure (both network models must remain open to the flow of matter and energy to be able to operate). In this regard, PM1 and PM2 differ in their external precursor demands: a difference that make them suitable for comparison as simplified models of a heterotrophic system (PM1) and of a relatively more elaborate, autotrophic one (PM2), respectively. Notice that the extra feedback reaction loop in PM2 allows the internal synthesis one of the precursors (AC), rendering the system less dependent on the availability of energy-rich precursors from the environment. In fact, one could think of PM2 as a developed form of PM1, resulting from an evolutionary bottleneck linked to the depletion of an environmental resource (AC), which would be consistent with a retrograde scenario for the evolutionary emergence of metabolism[4,5]. This comparative analysis is thus aimed at identifying possible dynamic constraints or advantages related with the early compartmentalization of such metabolic networks.

Modelling assumptions and parameter values

In order to facilitate direct comparison, PM1 and PM2 were simulated under similar rules and parameter conditions. All processes were considered reversible (favored in the direction pointed by the arrows in Fig. 2A-B), except for the reactions of decay that affect all catalysts, which were treated as irreversible (dashed arrows). Parameter values were arbitrary but consistently assigned. For simplicity, all unimolecular and bimolecular rate constants of the main core reactions (in dark colours) were given values equal to 1, using τ^{-1} and $mM^{-1}\ \tau^{-1}$ as default dimension units, respectively (τ = arbitrary units of time; see below). Conversely, the rate constants of those background processes corresponding to non-catalytic conversions (represented in light grey), which are assumed to occur more slowly, take values 1000 times smaller (i.e., 0.001, with the same units as before).

In the simplest modelling scenario, although no explicit considerations on the structure and dynamics of the compartment were included, a distinction was anyway made between the system's (internal) reaction domain (here considered as a fixed compartmentalized volume) and its surrounding environment. The concentrations inside the compartment were always considered variable, while the external concentrations of metabolic precursors were assumed as constant (i.e. buffered by external chemical sources) and equal to 1 mM throughout all the analyses.

The rates of exchange (uptake and release) of nutrients and end-products with the surrounding environment (through the membrane boundary), q_x, as well as the rate constants for the decay of the catalysts, i.e. k_4, k_8 (and k_{11} in PM2), were modified in order to explore the possible dynamic behaviours of the system. Being crucial parameters for the maintenance (or disintegration) of these active compartmentalized chemistries, they were consistently varied to assess the sensitivity/robustness of both network models (Fig. 2C-D). Steady-state concentration values for the different metabolic species were calculated by numerical solution of the set of ODEs describing their corresponding time courses. Chemical reaction dynamics were modelled assuming mass-action kinetics.

Permeability constraints

In the protometabolic models, all the external precursors adopt the form of activated monomers ('one-letter' species) except for the AC species, which is a more complex nutrient and serves to feed the simplest network system, the prototroph PM1. It is reasonable to assume that there is a penalty associated to this in the context of compartmentalized chemistries (like the one assumed in here): a bulkier precursor should in principle diffuse more slowly through a membranous boundary, into the reaction compartment. In consequence, the exchange rates for the A, B, C, D and w species were all set equal to each other and changed in concert (across simulations), whereas the AC exchange rate was assumed 200 times slower and changed accordingly, maintaining this ratio. Such a great difference in the permeation of larger oligomers is consistent with experimental observations on the selective permeability of model liposomes[6]. A related issue is the membrane leakiness to the internal metabolic intermediates. As a simplification, in our models oligomeric products were assumed to be totally impermeable, so their presence is circumscribed to the internal reaction medium. Indeed, *in vitro* assays demonstrate that lauric acid-based lipid vesicles are able to fully retain biopolymers (tRNA) for long periods of time, under experimental conditions similar to the ones used in this paper[7]. Nonetheless, the decay processes defined in the models, in principle intended to reflect the chemical lability and limited lifespan of the catalysts, could implicitly account also for a certain level of leakiness of the internal metabolic species.

An important first implication of the hindered accessibility of nutrients through the membrane compartment is the risk of collapse of encapsulated chemistries that take place too fast (self-suffocation)[5,8]. Reaction networks such as the ones analyzed here should occur at a relatively slow pace (at least in the range of hours) if they had to develop within prebiotic compartments like lauric-acid (LA) vesicles. This can be explained in the light of the steady-state diagrams shown in Fig. 2C-D, which are generalized for arbitrary units of time (τ). For example, a time scale of seconds

for the internal reactions would mean a requirement for nutrient uptake rates $q_x > 0.1\text{-}1\ s^{-1}$ in order to keep significant metabolite concentrations (Fig. 2C-D), a threshold that is far too high compared with the actual permeability values of LA vesicles ($\approx 3.1 \times 10^{-5}\ s^{-1}$, according to Table S2). Therefore, a time scale of hours for the protometabolic reaction rates was selected for all subsequent analyses (except otherwise stated). In terms of the catalyst decay rate constants, a value of $k_4 = k_8 = k_{11} = 0.05\ h^{-1}$ was generally used in the simulations. As a reference, experimental values of the rates of spontaneous hydrolysis of RNA oligomers fall within the same order of magnitude[9].

Membrane compartment and protocell modeling

In order to model realistically protocell dynamics, we used the computational platform ENVIRONMENT[10-12], which has been specifically designed to simulate stochastically the time behaviour of chemical systems encapsulated within dynamic lipid compartments. Using this simulation tool, a number of structural and dynamic features of the compartments can be specified, as summarized below (see also Fig. 2E). These include important physico-chemical constraints related with the topology and molecular composition of the enclosing membrane, which affect the internal metabolic processes and the overall space-temporal dynamics (for a more detailed description of the platform, refer to[11].

- *Compartment geometry:* The protocell compartment is delimited by a topologically closed membrane, a lipid bilayer of thickness λ that consists of a given amount of amphiphilic molecules (for cases in which there are only two types: $x_{l\mu}$ and $x_{L\mu}$) defining the actual surface area of the system S_μ. The protocell internal volume V_{in}, however, responds to osmotic differences between the inner and outer aqueous media, changing in order to re-establish the isotonic condition at each time step. In the analysis all soluble chemical species n (with molecular populations x_n) contribute to the osmotic tension. The decoupling of S_μ and V_{in} allows for different protocell shapes during the simulations, instead of keeping a perfect spherical geometry all the time (see below).

$$S_\mu = \frac{1}{2}\left(\alpha_l x_{l\mu} + \alpha_L x_{L\mu}\right)\ ;\ V_{in} = V_{out}\left(\sum_n^N x_{n,in} \Big/ \sum_n^N x_{n,out}\right)$$

  The parameters α_l and α_L refer to the polar head area of the integrating lipid species, l and L. We considered values equal to 0.2 nm^2 for LA, 0.4 nm^2 for GML, and 0.6 nm^2 for DLPC, in the range of experimental data[13]. In this study, all simulations started with a single spherical protocell of 100 nm diameter (i.e. $S_\mu = 3.14 \times 10^4\ nm^2$ and $V_{in} = 5.24 \times 10^5\ nm^3$), corresponding to a population of 314159 membrane amphiphiles in the case of a pure-LA membrane lipid composition ($\alpha_{LA} = 0.2\ nm^2$). We also assumed that $V_{out} >> V_{in}$, and V_{out} was actually kept constant ($V_{out} = 5.24 \times 10^8\ nm^3$) in all the simulations.

- *Membrane dynamics:* Membrane-integrating lipid species, l_μ and L_μ, were considered in fast dynamic equilibrium with their corresponding monomer forms, dissolved in the

aqueous medium (l_{in}/l_{out} and L_{in}/L_{out}). The processes of membrane lipid uptake and release were simulated for each amphiphile species, using as a first approximation the experimental values of CVC in the pure-composition systems as an estimate of the equilibrium aqueous concentrations of the individual lipids.[1] For LA, $[l_{aq}]_{eq} \approx 7.95$ mM, with a value of membrane uptake rate $k_{l\mu} \approx 7.6 \times 10^{3}$ M^{-1} nm^{-2} s^{-1} and a molecular release constant $k_l \approx 6.042$ s^{-1}, as inferred from[11]. However, these rates fall too high compared with the timescale of the other protocellular processes, so in practice, for most long-term-behaviour simulations, the lipid exchange parameters were rescaled to lower values (i.e. $k_{l\mu} \approx 7.6$ M^{-1} nm^{-2} h^{-1}; $k_l \approx 6.042 \times 10^{-3}$ h^{-1}), which still ensured a rapid lipid dynamic equilibrium and gave similar phenomenological outcomes.

- *Molecular permeability across the membrane:* Each of the small metabolic species (nutrients or side-products) that is assumed to freely diffuse across the membrane (see "Permeability constraints" within the "Protometabolic network modelling" section above) was assigned a molecular diffusion rate, D_x, that is a function of the membrane lipid composition. Experimental values of membrane solute permeability, k_x (and P_x), were translated into molecular diffusion rates, as detailed elsewhere[8] (Fig. 1; Table S2), considering the approximate liposome sizes and the hypothetical membrane thickness (radius $r \approx 200$ nm, $\lambda \approx 3$ nm in all membrane models considered here). The actual curves of molecular diffusion (which are dependent on the lipid molar ratio: see Fig. 1B) were fitted with exact polynomials of the form $ax^4 + bx^3 + cx^2 + dx + e$, which were then implemented for the simulation of the transport processes in the binary-lipid protocell models, except when stated otherwise (a = -1.6, b = 4.2, c = -3.6, d = 1.0, e = 0.0038 ($\times 10^{8}$ dm^2 mol^{-1} s^{-1}) for LA/DLPC; a = 0, b = 2.0, c = -3.6, d = 1.4, e = 0.0038 ($\times 10^{8}$ dm^2 mol^{-1} s^{-1}) for LA/GML). We thus simply assumed that all small diffusing solutes in the model respond in the same way and show permeability properties similar to those displayed by carboxyfluoresceine (CF) in the *in vitro* assays. Regarding the permeability to the AC species, the same simplifications (as in previous sections -- see "Permeability constraints") hold. Most simulations were initiated with a pure-l lipid membrane and metabolite concentrations at the steady-state levels corresponding to that condition, i.e. $D_x = 3.8 \times 10^{5}$ dm^2 mol^{-1} s^{-1}, for x = A, B, …, w (see Fig. 2C-D as a reference of the steady-state value dependence in terms of exchange rates $q_x \approx k_x$).

- *Structural stability:* The geometrical shape of a protocell defines its structural stability. Insofar as a protocell can abandon the spherical shape, considered as the minimum energy state, it may adopt swollen or deflated forms. Hence the convenience of defining a stability factor, Φ, usually called the 'reduced surface', which relates the actual protocell surface area S_μ with that of a perfect (theoretical) sphere containing its actual volume V_{in}:

[1] We are aware, though, that the scenario with a mixture of lipids may be more complicated, with CVC values (and membrane lipid exchange rates) that vary nonlinearly with changes in the molar ratios[14]. This is an area that requires further exploration in future work.

$$\Phi = \frac{S_\mu}{\sqrt[3]{36\pi {V_{in}}^2}} \quad ; \quad (1-\varepsilon) \le \Phi \le \sqrt[3]{2}$$

For a spherical cell, Φ = 1. But Φ can adopt different values. If the cell is markedly deflated ($\Phi > \sqrt[3]{2}$), it divides to give two spherical daughter cells of half the actual volume. Similarly, if it is excessively swollen, Φ < (1- ε), it suffers an osmotic burst. An (invariant) osmotic tolerance of ε = 0.21 was considered in all the simulations[11].

Although ENVIRONMENT in principle allows keeping track of the stochastic time evolution of each and all of the daughter cells generated upon successive divisions, such a comprehensive implementation turns out to be quite time-consuming, computationally speaking. In the current work, only a single cell, the "mother-cell" lineage, was therefore followed over time. The protocell population behaviour, wherever applicable, was inferred from the time evolution and events occurring in this reference cell. Such an approach is suitable insofar as the two generated daughter cells are considered statistically identical (i.e., having same state variables). Although one cannot account for asynchronous division events, the platform is still able to recapitulate the statistical behaviour of the successive division times, by carrying out multiple independent simulations. Convergence with the average population behaviour was assured: the models were alternatively implemented in MATLAB and analyzed deterministically, giving as an outcome similar time courses, both for the size of the cell population, as well as in terms of cellular attributes (data not shown).

Considerations about lipid production and the osmotic control on protocell size

Two crucial requisites have to be fulfilled by these protocell systems, if they are to achieve stationary reproduction regimes[12]: they have to ensure (i) a sustained lipid production, and (ii) a net accumulation of an osmotically-active species inside the compartment. Regarding the lipid production, an internal process of synthesis of a lipid species L was considered in our model, catalyzed by the most abundant metabolic intermediate AB (both in the PM1 and the PM2 cases -- Fig. S5), from some precursor P, which was available in the environment (Fig. 2E). The external concentration of this precursor was fixed ($[P]_{out}$ = 0.5 mM, unless otherwise stated) and P was assumed to diffuse through the membrane like any other small precursor (i.e. with the same permeability values as explained above). For the production rate constant, k_S, was set to 1 mM^{-1} h^{-1}. Additionally, a lipid decay process was included in the models to account for the labile nature of the internally synthesized (more complex) lipid L, as well as to favor stationary conditions of intermediate, heterogeneous lipid compositions (see Fig. 2E). For simplicity, L → 2l, with a tunable decay rate constant k_d that controlled the degree of conversion (relative abundance of both lipids) in the membrane composition (Fig. S8). Given the stoichiometry of this reaction and the realistic values of the different lipid polar-head areas (α_l and α_L, as assigned above), someone may argue that increased values of k_d could lead to a certain delay in the vesicular growth dynamics. However, we checked that this is a negligible effect compared with the impact from membrane permeability changes, as can be derived from Fig. S8.

In terms of the osmotic tension, in addition to all osmotically-active molecules commented so far, the presence of a buffering species, B, was included at both sides of the membrane (it was considered relatively abundant: [B] in the range of 50 mM). As shown in previous work[10,11] and confirmed here (data not shown), the inclusion of this chemically inactive osmolite contributes to stabilize the compartment by reducing the random osmotic fluctuations and mimics adequately the activity of the buffering species, bicine in our case, in the *in vitro* models. With these assumptions, in a first exploratory analysis, we tried models where all metabolic end-products consisted of non-active monomers w (as depicted in Fig. 2A-B), a species that would passively diffuse out of the compartment. Although these models allowed a transient accumulation of species w, the internal levels of the waste product reached equilibrium, since w molecules were released to the external medium. It followed that a number of protocell divisions took place, with a progressive shrinkage of the protocell sizes, until the full lineage collapsed. Decreasing the membrane permeability coefficient of the waste species didn't solve the problem, but just delayed the entry into this 'deadly' division regime: the w species evolved to higher equilibrium concentration values in the internal medium, after which no "extra" osmotic tension was generated (data not shown). In order to circumvent this difficulty, we had to consider at least one of the end-products of metabolism as an impermeable species, i.e. a non-active, degraded oligomer (in this case acd, derived from ACD). With this setup and under considerations similar to those described above, we found conditions for stationary reproduction under a strikingly wide range of parameter values with both models, PM1 and PM2 (Fig. S7), suggesting an ample robustness of the stationary reproduction regime under these assumptions.

References

1. Rosen, R. *Life itself: a comprehensive inquiry into the nature, origin and fabrication of life.* (Columbia University Press, 1991).

2. Piedrafita, G., Montero, F., Morán, F., Cárdenas, M.L. & Cornish-Bowden, A. A simple self-maintaining metabolic system: robustness, autocatalysis, bistability. *PLoS Comput Biol* **6,** e1000872 (2010).

3. Ruiz-Mirazo, K. & Moreno, A. Basic autonomy as a fundamental step in the synthesis of life. *Artif Life* **10,** 235–259 (2004).

4. Horowitz, N.H. On the Evolution of Biochemical Syntheses. *Proc Natl Acad Sci U S A* **31,** 153–7 (1945).

5. Szathmáry, E. Coevolution of metabolic networks and membranes: the scenario of progressive sequestration. *Philos Trans R Soc Lond B Biol Sci* **362,** 1781–7 (2007).

6. Monnard, P.A. & Deamer, D.W. Nutrient uptake by protocells: a liposome model system. *Orig Life Evol Biosph* **31,** 147–55 (2001).

7. Maurer, S.E., Deamer, D.W., Boncella, J.M. & Monnard, P.-A. Chemical evolution of amphiphiles: glycerol monoacyl derivatives stabilize plausible prebiotic membranes. *Astrobiology* **9,** 979–87 (2009).

8. Piedrafita, G., Ruiz-Mirazo, K., Monnard, P.-A., Cornish-Bowden, A. & Montero, F. Viability conditions for a compartmentalized protometabolic system: a semi-empirical approach. *PLoS One* **7,** e39480 (2012).

9. Adamala, K. & Szostak, J.W. Nonenzymatic template-directed RNA synthesis inside model protocells. *Science* **342,** 1098–100 (2013).

10. Mavelli, F. & Ruiz-Mirazo, K. Stochastic simulations of minimal self-reproducing cellular systems. *Philos Trans R Soc Lond B Biol Sci* **362,** 1789–802 (2007).

11. Mavelli, F. & Ruiz-Mirazo, K. ENVIRONMENT: a computational platform to stochastically simulate reacting and self-reproducing lipid compartments. *Phys Biol* **7,** 036002 (2010).

12. Mavelli, F. & Ruiz-Mirazo, K. Theoretical conditions for the stationary reproduction of model protocells. *Integr Biol* **5,** 324–41 (2013).

13. Kucerka, N. *et al.* Structure of fully hydrated fluid phase DMPC and DLPC lipid bilayers using X-ray scattering from oriented multilamellar arrays and from unilamellar vesicles. *Biophys J* **88,** 2626–37 (2005).

14. Shirt-Ediss, B., Ruiz-Mirazo, K., Mavelli, F. & Solé, R.V. Modelling lipid competition dynamics in heterogeneous protocell populations. *Sci Rep* **4,** 5675 (2014).

Table S1. CVC values obtained for different binary lipid mixtures at 45°C.

	Lipid molar ratio	pH	CVC (mM) *
LA/GML	1:0	8.00	7.95 ± 0.68
	4:1	8.05	0.29 ± 0.04
	2:1	8.10	0.17 ± 0.01
	1:1	8.70	0.10 ± 0.02
	1:2	9.60	0.07 ± 0.05
LA/DLPC	1:0	8.00	7.95 ± 0.68
	3:1	8.00	0.03†
	1:1	8.00	0.02†
	0:1	8.00	0.02†

* Values from N ≥ 3, presented as mean ± SD.

† Values coming from a single experiment.

Table S2. Permeability coefficients of different mixed-composition lipid vesicles at 45°C.

Amphiphile composition	Total conc. (mM)	pH	k_x (x10^{-5} s^{-1}) *	P_x (x10^{-10} cm/s) *	D_x (x10^{5} dm^{2}mol^{-1}s^{-1}) *
LA	21	8.00	3.1 (2.5;4.0)	2.1 (1.7;2.7)	3.8 (3.1;4.8)
LA/LOH 10:1	30	8.50	10.1 (9.3;10.8)	0.8 (0.8;0.9)	1.5 (1.4;1.6)
OA/GMO 2:1	30	9.10	0.4†	0.03†	0.08†
LA/GML 1:0	21	8.00	3.1 (2.5;4.0)	2.1 (1.7;2.7)	3.8 (3.1;4.8)
LA/GML 4:1	17	8.00	134.0 (133.0;141.0)	89.3 (88.7;94.0)	161.4 (160.2;169.8)
LA/GML 2:1	12	8.00	127.0 (66.6;153.0)	84.7 (44.4;102.0)	153.0 (80.2;184.3)
LA/GML 1:1	12	8.00	52.8 (47.8;57.7)	35.2 (31.9;38.5)	63.5 (57.6;69.5)
LA/DLPC 1:0	21	8.00	3.1 (2.5;4.0)	2.1 (1.7;2.7)	3.8 (3.1;4.8)
LA/DLPC 3:1	10	8.00	75.7 (57.0;84.3)	50.5 (38.0;56.2)	91.2 (68.7;101.5)
LA/DLPC 1:1	7	8.00	31.6 (24.0;49.5)	21.1 (16.0;33.0)	38.1 (28.9;59.6)
LA/DLPC 1:3	6	8.00	7.1 (6.6;8.1)	4.7 (4.4;5.4)	8.5 (7.9;9.8)
LA/DLPC 0:1	3	8.00	10.8 (9.7;12.1)	7.2 (6.5;8.1)	13.0 (11.7;14.6)

* Values from $N \geq 3$, presented as median (quantile 1; quantile 3).
† Values for the OA/GMO mixture come from a single experiment.

Fig. S1. Experimental procedure and validation controls of vesicle formation and solute encapsulation. (A) Protocol used for vesicular encapsulation and permeability measurements. pH vesiculation was performed at temperatures over 43°C (the melting point of LA) and then suspensions were kept at 45°C, well above the transition temperature of LA membrane bilayers (T_m ≈ 32°C), throughout the experiments. (B) Epifluorescence micrographs showing LA/GML vesicles (lipid ratio 2:1) after the pH-induced vesiculation method (upper panel; membranes stained with the lipophilic dye Nile red) and just after the size-exclusion chromatography (middle panel; retained carboxyfluoresceine, CF, in green). Membrane solubilisation with Triton-X100 forced the complete homogenization of CF in the sample, with no visible vesicular structures (bottom panel). (C) Absorbance pattern of the different elution samples collected from the size-exclusion chromatography, confirming the adequate separation of the non-entrapped solute (CF detected at 450 nm: black trend; signal at 650 nm – grey trend -- indicates vesicle scattering). Vesicles co-eluted with the first peak of CF. (D) Calibration curve of CF fluorescence signal (450 nm), used to convert the time evolution of the fluorescence intensity into concentration time courses in the solute-release experiments (notice that CF self-quenches over 0.1 mM). (E) CF release curves obtained at 45°C with two representative types of lipid vesicles: pure LA vesicles, and LA/GML (2:1) vesicles (that resulted more permeable). 2-3 small aliquots of Triton-X100 detergent were eventually added to ensure complete equilibration of CF ($[CF]_{eq}$ value).

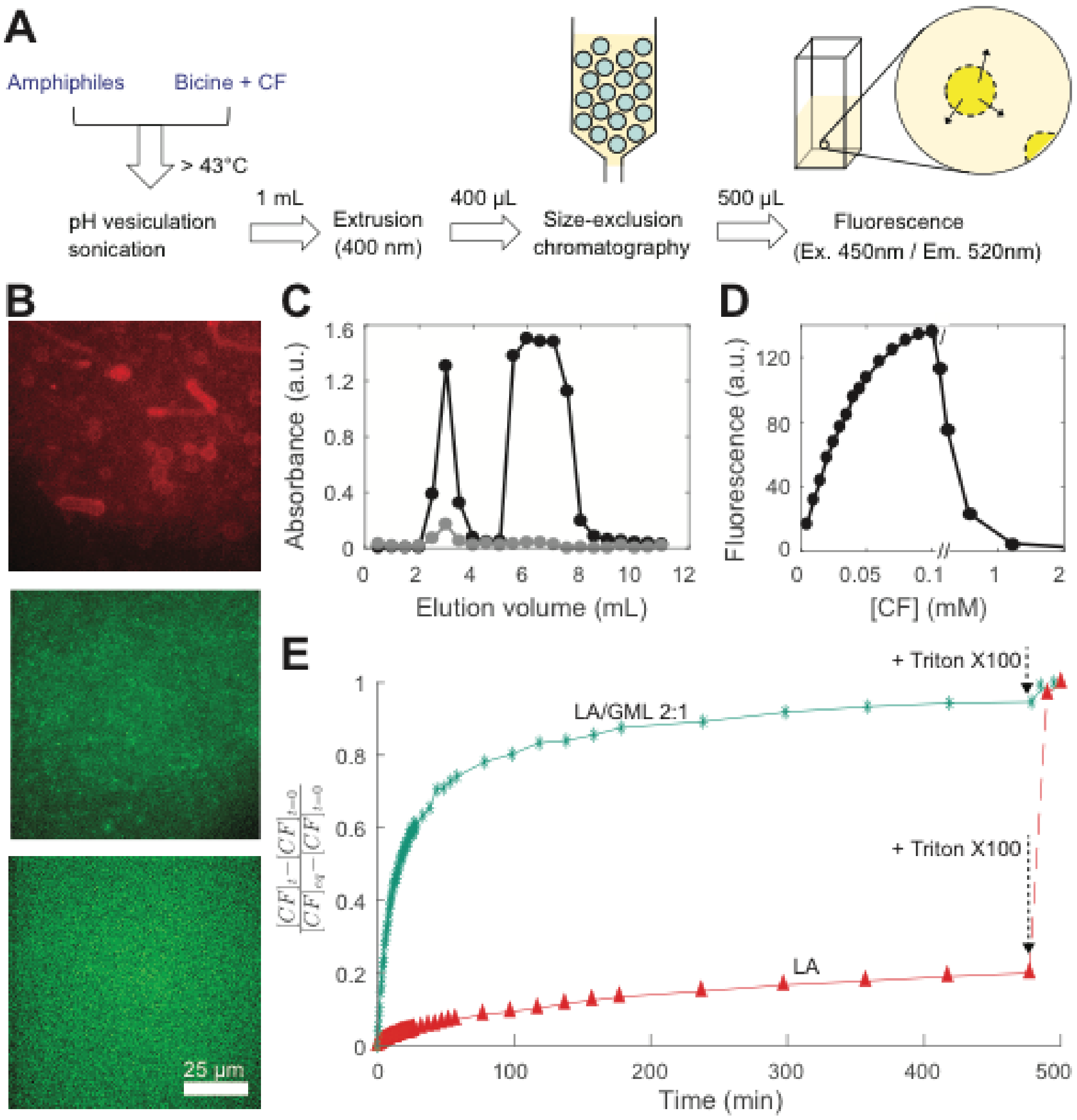

Fig. S2. Trends of solute retention inside different types of lipid vesicles at 45 ˚C. The percentage of initial entrapped carboxyfluoresceine (CF) that remains encapsulated at various incubation times was derived from long-duration release assays. A solubilising detergent was used for final equilibration as a reference (0% encapsulation). (A) Values obtained with LA (and a 10:1 mixture with the corresponding alcohol) as compared to LA/GML vesicles. Higher relative amounts of GML did not yield sufficiently stable vesicles. (B) Values obtained with mixed LA/DLPC vs. pure LA and pure DLPC vesicles. Data shown as mean ± SD (N ≥ 3).

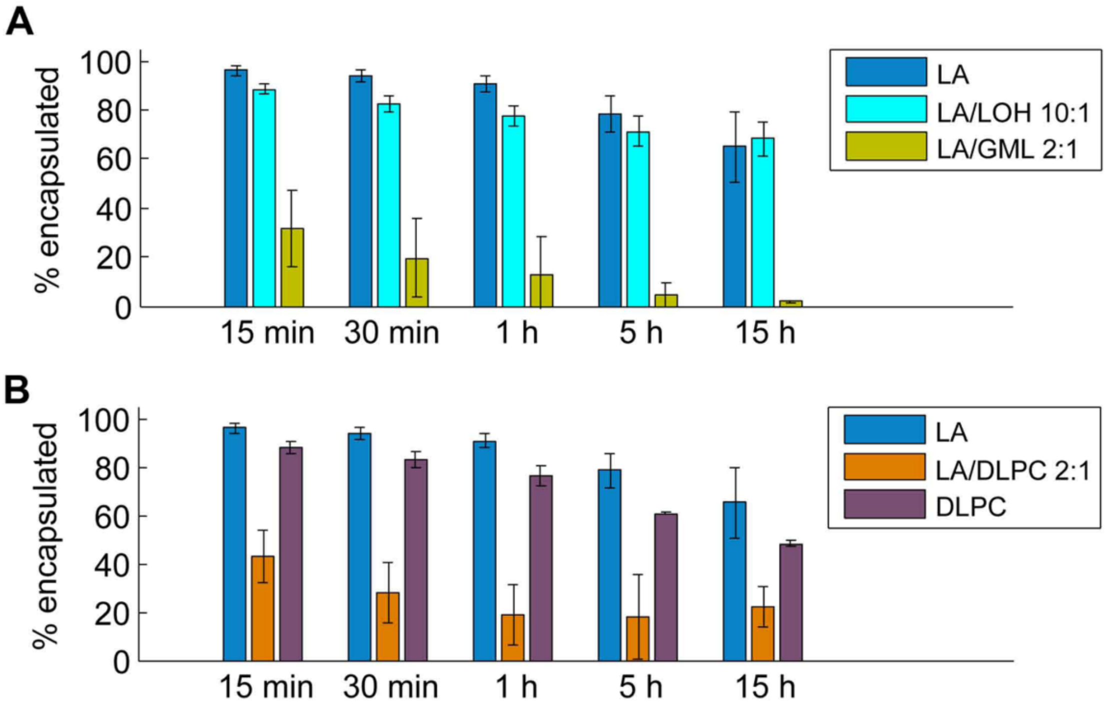

Fig. S3. Calculation of the CVC values for two representative lipid systems at 45ºC, using the merocyanine-540 method. The absorbance ratio $Abs_{570\,nm}$ / $Abs_{530\,nm}$ obtained for different serial dilutions of vesicle suspensions made with LA alone (A) or LA/GML in a molar ratio 4:1 (B) is shown. The total lipid concentration where the inflexion point was detected was identified as the cvc value -- significantly lower in the mixture (LA/GML 4:1) than in the pure LA case. For the complete set of values, see Table S1.

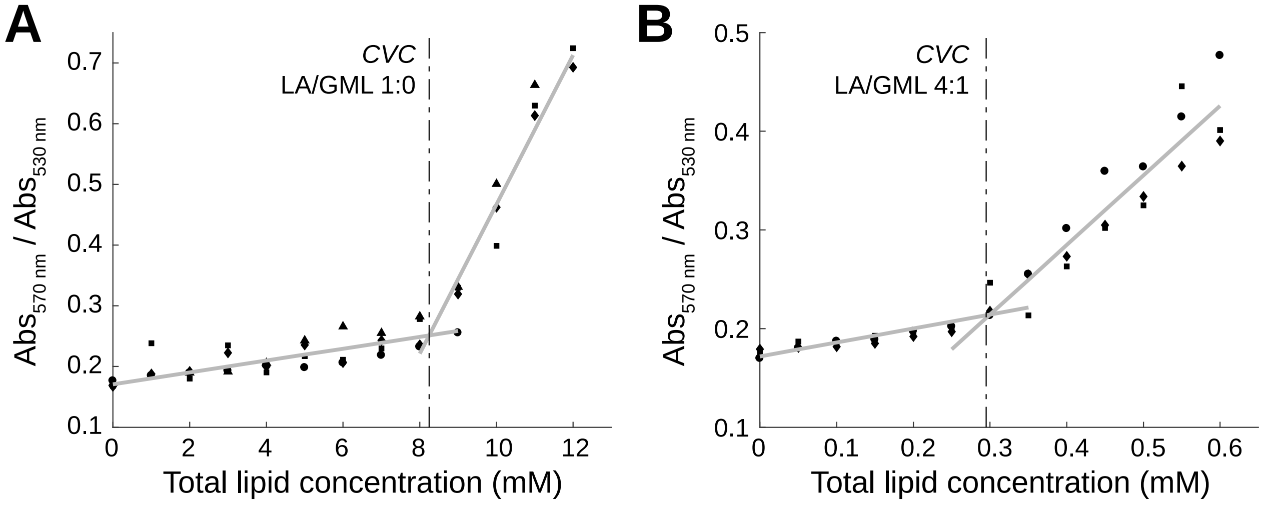

Fig. S4. Vesicle aggregation and stabilization as a function of pH and lipid composition. (A) Phase transitions revealed by light scattering for diverse aqueous dispersions of LA/GML at different molar ratios (in all cases, 60 mM total amphiphile concentration; experiments performed at 45°C). The progressive enrichment in GML expands the pH range in which vesicle aggregation takes place towards alkaline conditions, as indicated by the increased light scattering. (B) Titration curves obtained from the experiments in (A) (non-buffered conditions). The inflexion point in the purple curve corresponds with the apparent pK_a of LA at 45°C ($pK_a \approx 8.00$).

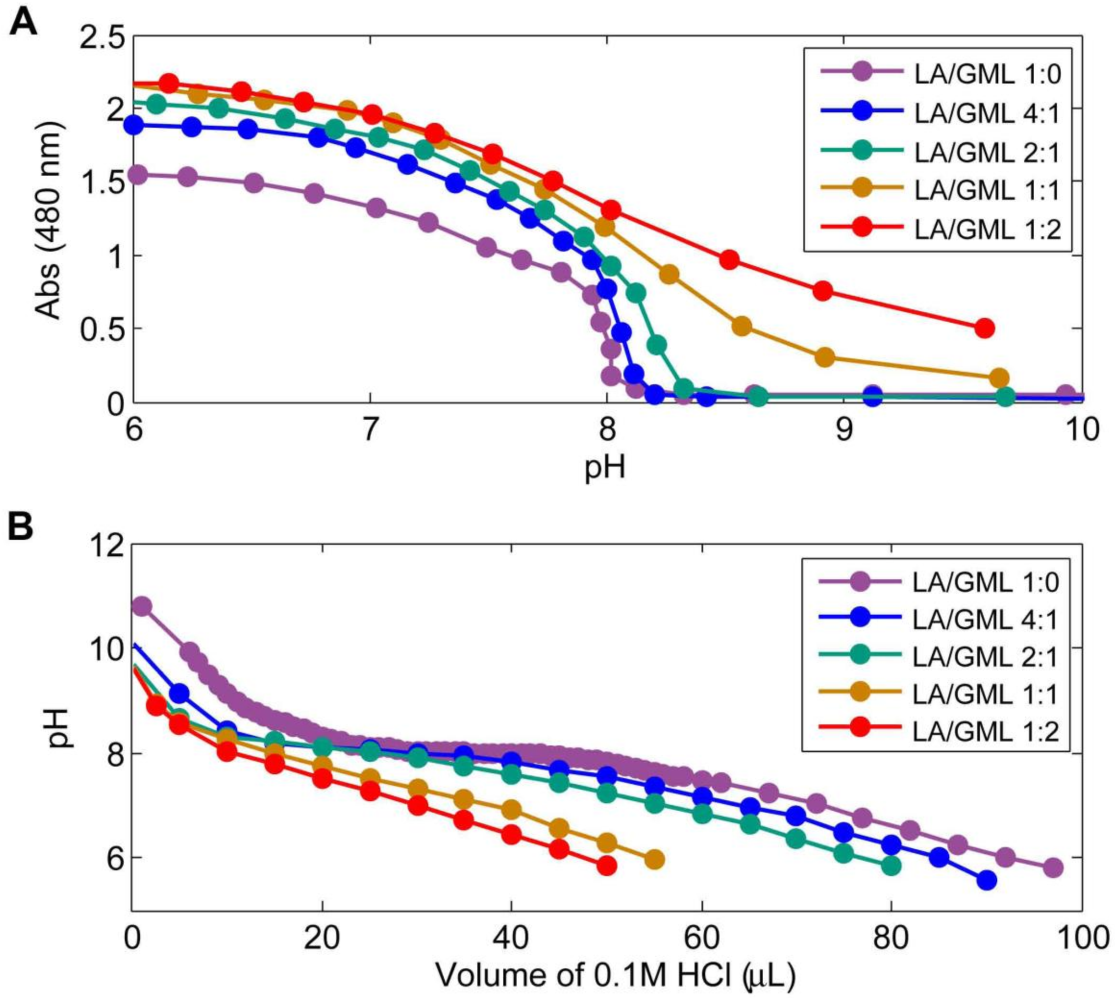

Fig. S5. Concentration levels of protometabolic intermediates prior to and upon encapsulation inside model protocells. Membrane permeability constraints hinder the accessibility of nutrients to the internal aqueous medium, leading to a more limited development of the encapsulated proto-metabolisms in comparison to free, aqueous-solution conditions ($q_x \rightarrow \infty$; constant supply of nutrients). Yet, metabolic species persist with concentration levels fluctuating around non-null steady-state values. Membrane permeability values were assumed to be equal to those described for LA vesicles -- for results with other lipid compositions, refer to Fig. 4A-B (note that the "total content" reported in that figure includes the internal concentration of precursors, too). (A) Results from the PM1 model. (B) PM2 model. The stochastic probability distribution function obtained for each intermediate is shown on the right panel.

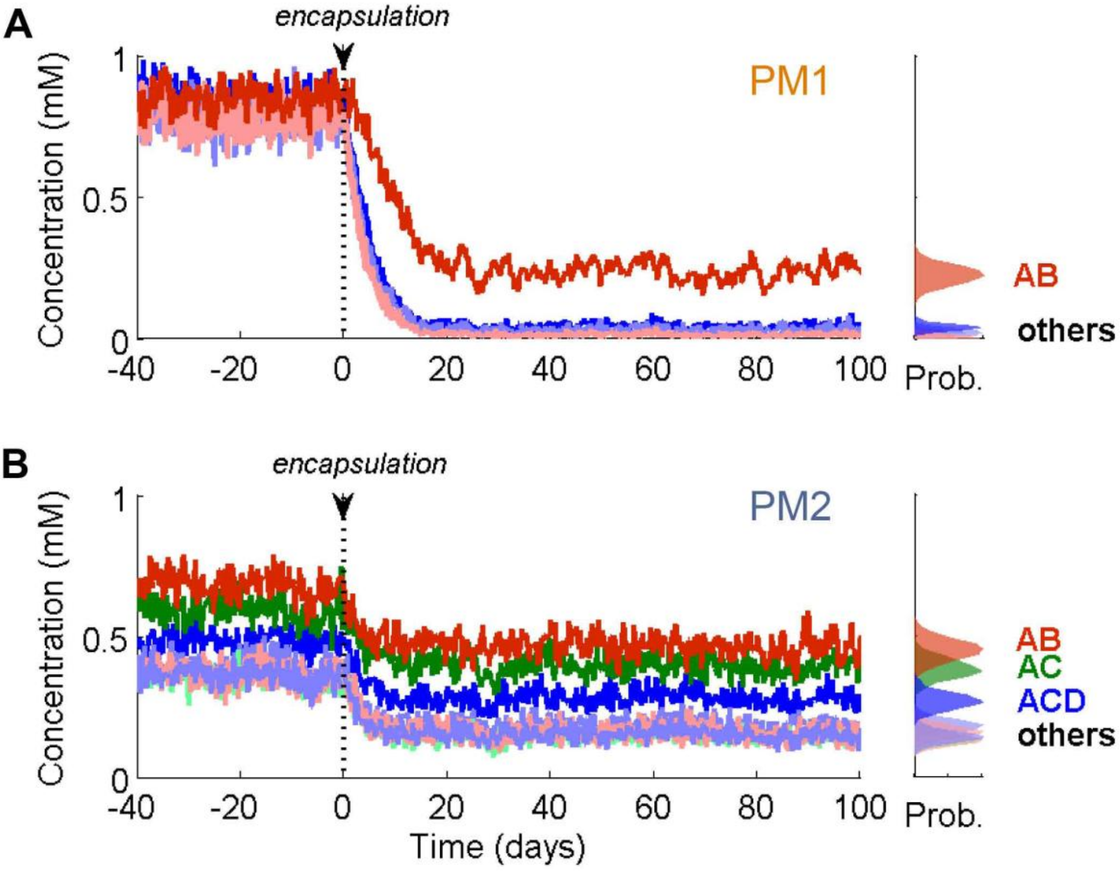

Fig. S6. Implementation of the lipid composition-dependent membrane permeability for protocell dynamics modelling. For validation purposes, the simple scenario of a single-nutrient uptake by empty vesicles of different lipid ratios was simulated, considering the empirical non-linear functions of Fig. 1B (main text). (A) Influx from a 10 mM stock of solute occurs faster for heterogeneous membranes, both in LA/GML and LA/DLPC systems, with equilibration taking place within 1 h. (B) Protocellular changes in a LA/DLPC 3:1 vesicle following nutrient uptake. As the solute accumulates internally, it osmotically generates an increase of the vesicular volume. This leads to a swollen-shaped structure (lower values of parameter Φ) with invariant membrane surface area. The relative proportions of both lipid species in the membrane do not significantly change (ratio 3:1), and their concentrations in the aqueous medium keep fluctuating around equilibrium values related with their corresponding CVC values.

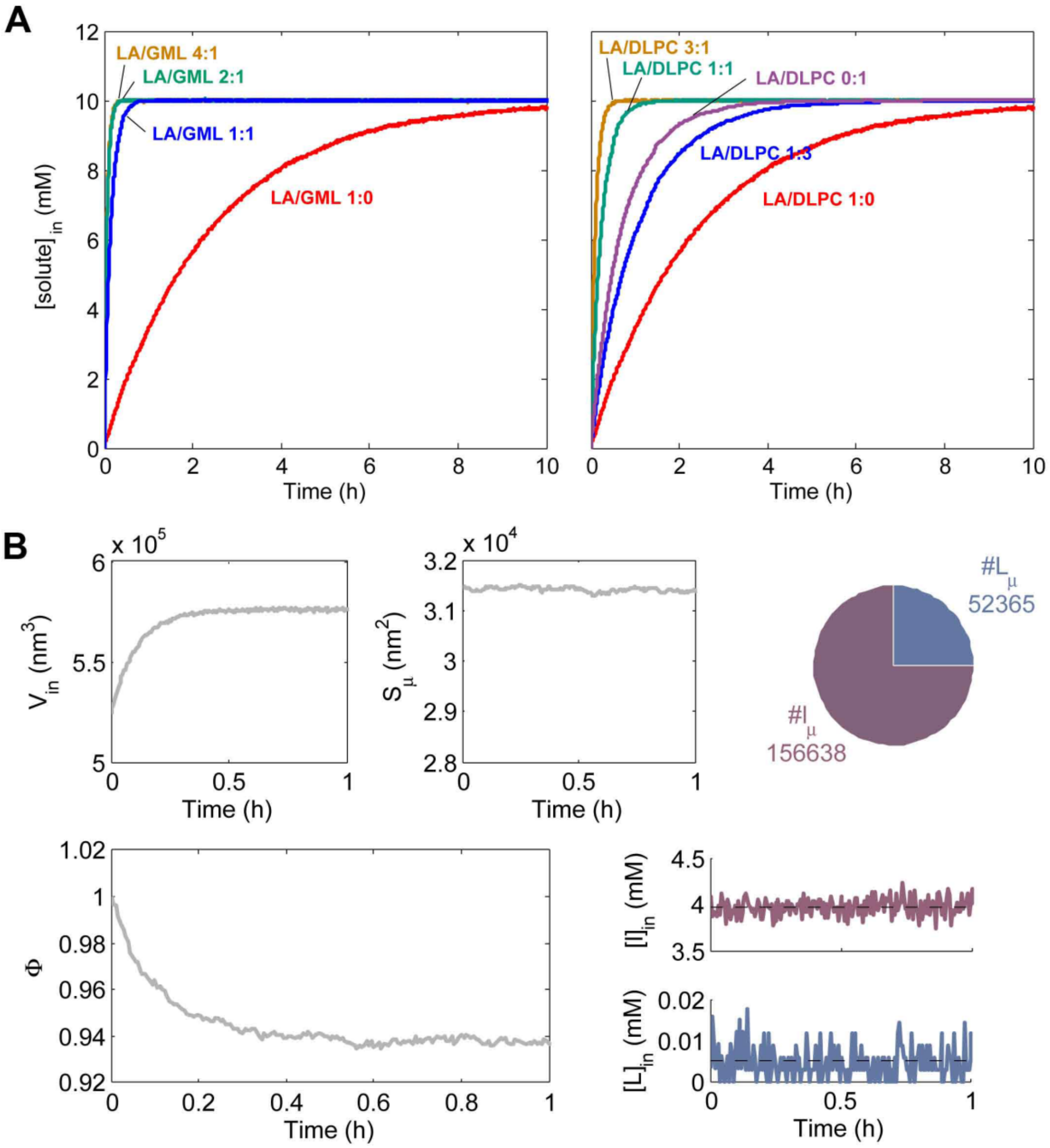

Fig. S7. Robustness of the stationary reproduction regime against perturbations on different variables/features related to protocell function. (A) The global speed of the internal metabolic processes was altered, setting all individual rates to a time scale of (i) weeks, (ii) days, or (iii) hours. (B) Different availability conditions (external concentrations) of the lipid precursor P were considered: $[P]_{out}$ = 0.5 (i), 1 (ii), and 2 (iii) mM. (C) The permeability values to the end-product species (w) were selectively manipulated to be equal (i), or 4 (ii), 10 (iii), or 100 (iv) times smaller than those for nutrient species. Only in the latter case the massive internal accumulation of w led to an osmotic burst (Φ < 0.79). (D) The physical-chemical properties of the internally synthesized lipid species were modifiable, adopting similar features as LA (i), GML (ii), or DLPC (iii). The PM2 scheme was considered in all cases. Regardless of the parameter affected, a stationary division regime was the normal outcome.

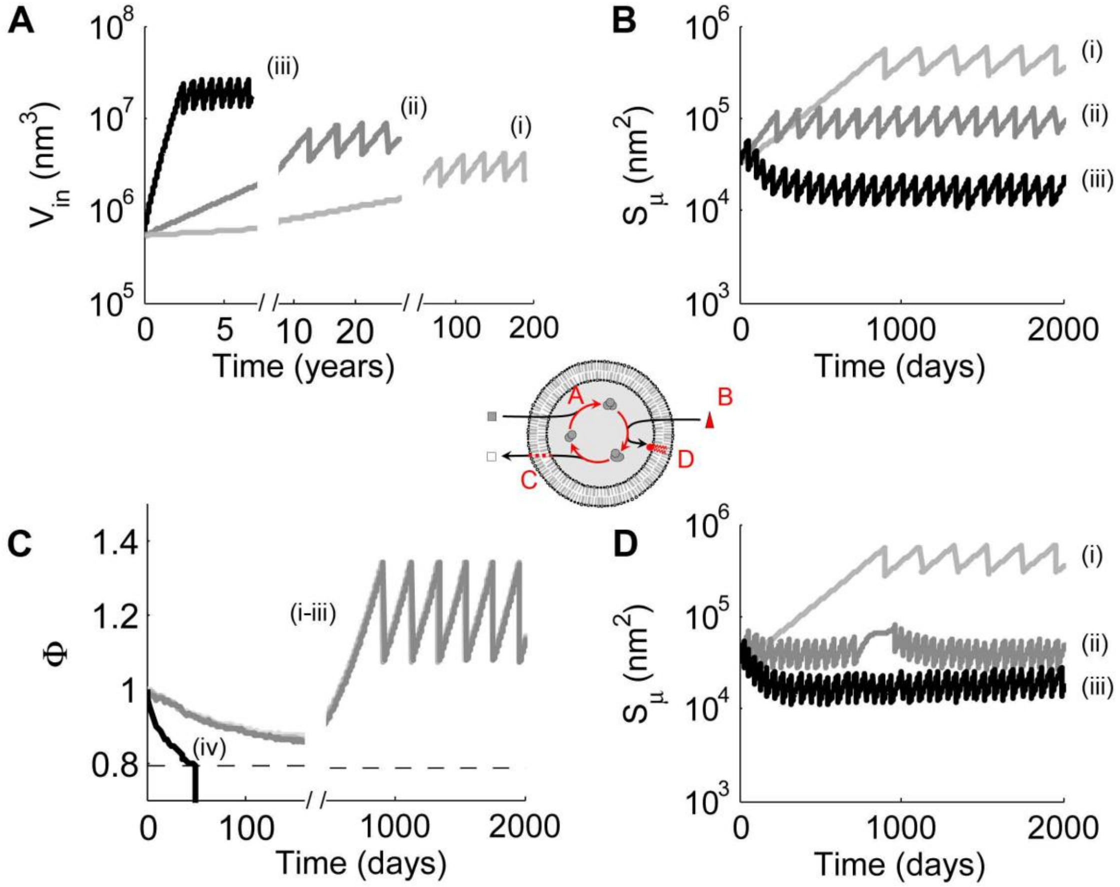

Fig. S8. Time evolution of the lipid composition of the membrane with diverse metabolic rates of lipid synthesis, and the corresponding protocell-division regimes. (Left) Lipid abundances are shown as the membrane relative surface fraction covered by the LA species, χ_S^I, and by the DLPC species, χ_S^L (black and grey lines, respectively). Simulations were carried out with the PM2 model, varying the value of the parameter k_d: (A) 0.01, (B) 0.002, (C) 0.0005, (D) 0 h^{-1}. (Right) Simulated protocells undergo multiple cycles of division, as reflected in the common jagged pattern shown for the stability parameter Φ over time, with differences in the stationary division time (values calculated as mean ± SD from 10 consecutive late generations, t > 2000 days).

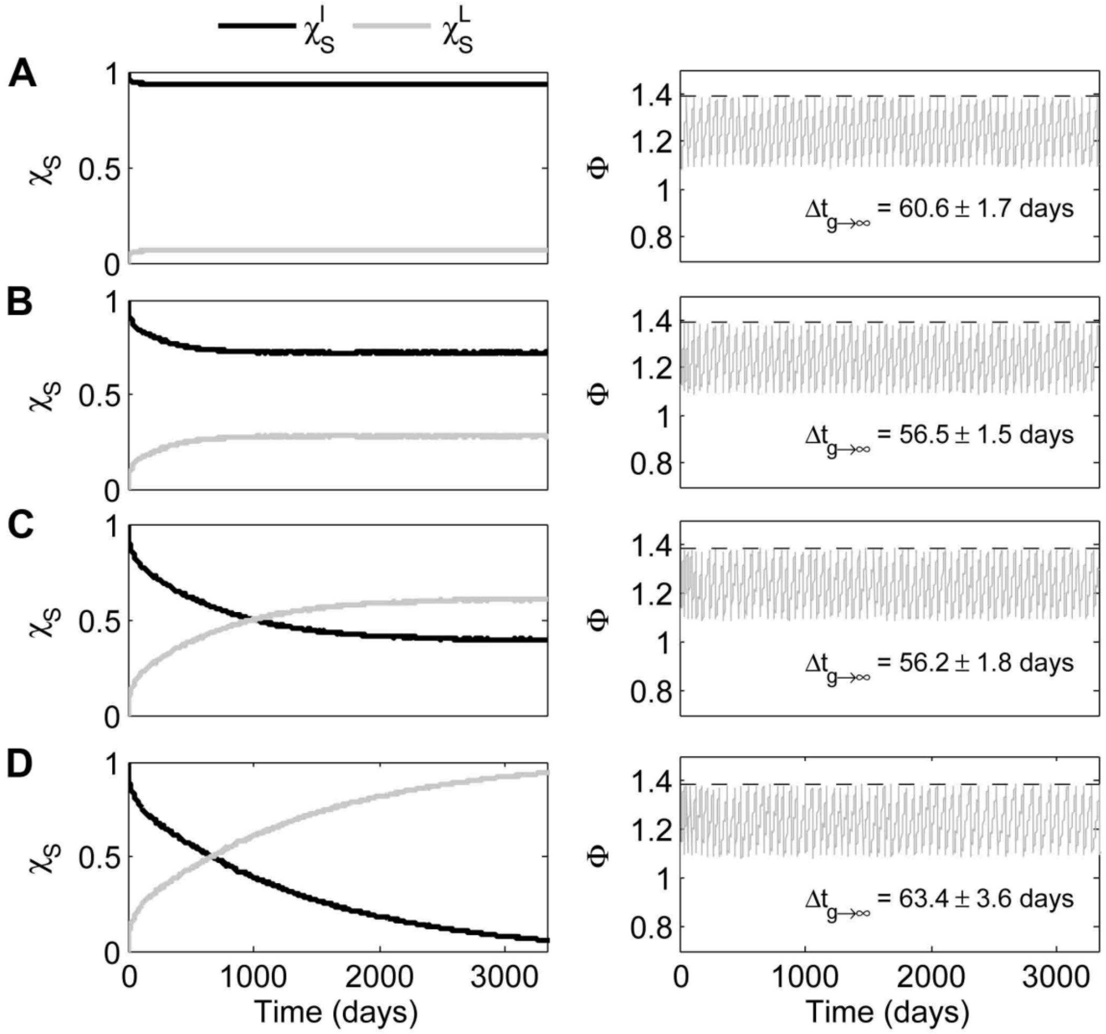

Supplement: Supplementary file 1 — SUPPLEMENTARY_INFO_PIEDRAFITA_ET_AL [file 41598_2017_2799_MOESM1_ESM.pdf]
